# The COM-B model: a cross-sectional survey assessing capability, opportunities, and motivation to follow the MIND diet among informal female caregivers of people with Alzheimer's disease and related dementias

**DOI:** 10.3389/frdem.2024.1451310

**Published:** 2024-10-03

**Authors:** Jacqueline Guzman, Susan Aguiñaga

**Affiliations:** ^1^Cancer Center, Medical College of Wisconsin, Milwaukee, WI, United States; ^2^Department of Health and Kinesiology, University of Illinois Urbana-Champaign, Champaign, IL, United States

**Keywords:** informal caregiving, dietary patterns, brain health, dementia care, caregiver nutrition

## Abstract

**Introduction:**

Caring for a person with Alzheimer's disease or dementia has been correlated with poor dietary patterns in caregivers. Dietary patterns like The Mediterranean-DASH diet intervention for neurodegenerative delay (MIND) diet have the potential to reduce the negative health outcomes associated with caregiving. Our objective was to assess capabilities, opportunities, and motivation of caregivers to follow the MIND diet using the COM-B model approach.

**Method:**

Female caregivers (*n* = 299, *m*_age_ = 37.7 ± 13.7) participated in an online survey. Majority were White (72%) and cared for someone with Alzheimer's disease (42.6%). The survey included at least one question for each of the 6 COM-B subcomponents: psychological capability, physical capability, social opportunity, physical opportunity, reflective motivation, and automatic motivation.

**Results:**

Most caregivers were not consuming the MIND diet as only 8.4% reported normally eating the MIND diet items. Caregivers (36.5%) were slightly confident or not confident at all in cooking and eating the MIND diet. Participants (67.1%) reported that consuming the MIND diet would somewhat to very much be supported by friends and family. Budget, time, and transportation were selected as the main barriers. Budget, cooking skills, access to food and stores, and family support were the main facilitators.

**Discussion:**

Strategies to increase capability, opportunities, and motivation for the MIND diet are needed to improve caregivers' health. Future MIND diet interventions should improve budget planning and cooking skills of caregivers (capabilities), make MIND diet food items accessible to them (opportunity) and incorporate social support from family and friends (motivation).

## Introduction

Millions of Americans provide care for an older adult including providing care for family members or friends with Alzheimer's disease and related dementias (ADRD; Alzheimer's Association, 2023). Specifically, over 11 million women in the U.S either live with or care for someone with ADRD (Alzheimer's Association, 2023). Compared to any other caregivers, caregivers of people with ADRD experience more challenging, more intense, and longer-term caregiving situations (Alzheimer's Association, 2023; Kapoor et al., 2020; Ory et al., 1999). According to the Alzheimer's Association, on average, caregivers of people with ADRD provide more care hours than non-ADRD caregivers because people with ADRD require higher levels of care related to activities of daily living and instrumental activities of daily living and exhibit more behavioral problems (Alzheimer's Association, 2023). Often, caregivers forgo their own health for those they care for. Caregivers of people with ADRD experience higher levels of stress and depression compared to other caregivers (Watson et al., 2019). They also have higher prevalence of chronic conditions including hypertension, arthritis, and heart disease (Wang et al., 2014). Additionally, they have been shown to have stress-related cognitive dysfunction (Allen et al., 2017; Correa et al., 2016; Oken et al., 2011), increased risk for anxiety (Watson et al., 2019), poor sleep quality (Gao et al., 2019; Cupidi et al., 2012), poorer quality of life (Andreakou et al., 2016), and poorer health behaviors (Vitaliano et al., 2003).

Poor health behaviors include poor dietary patterns that can further negatively impact health. About 15% of caregivers report eating no more than two meals per day (Rabinowitz et al., 2007), and othersreport eating unhealthy foods including fast food due to the lack of time or as a coping mechanism (Wang et al., 2019). In a study by Rullier et al. (2013) it was found that 32.1% of family caregivers of people with ADRD were at risk of malnutrition and 5.4% of the caregivers were malnourished. Dietary patterns like the Mediterranean-DASH diet intervention for neurodegenerative delay (MIND) diet have the potential to reduce negative health outcomes associated with caregiving, unfortunately this has not been explored in caregivers of people with ADRD.

The MIND diet is a dietary pattern that is tailored for neuroprotection including the most compelling evidence in the diet-dementia research. It is a combination of the Mediterranean diet and the Dietary Approaches to Stop Hypertension (DASH) diet. The MIND diet consists of 10 brain healthy food groups (green leafy vegetables, other vegetables, nuts, berries, beans, whole grains, fish, poultry, olive oil, and wine [1 glass/d]) (Morris et al., 2015). For safety, the wine recommendation was removed in the MIND diet trial (Liu et al., 2021). The MIND diet also recommends limited intake of 5 food groups (high saturated fat or sugar intakes, such as red meat and meat products, butter, whole fat cheese, pastries and sweets and fried and fast foods) (Morris et al., 2015). Following the MIND diet might help caregivers alleviate some of the negative effects of caregiving. Previous studies in non-caregivers have shown association between the MIND diet and fewer depressive symptoms (Cherian et al., 2021), reduced risk of cognitive impairment (Morris et al., 2015) and improved sleep (Campanini et al., 2017; Castro-Diehl et al., 2018; Godos et al., 2019; Mamalaki et al., 2018; Zuraikat et al., 2020).

Few studies have explored barriers and facilitators of adopting healthy dietary patterns like the MIND diet including the Mediterranean and the DASH diets. For instance, a systematic review by Tsofliou et al. (2022) examined barriers and facilitators to the Mediterranean diet in both Mediterranean and non-Mediterranean countries. They found that barriers included lack of knowledge or misconceptions about the diet, cultural preferences and traditions, busy lifestyles, and limited availability of Mediterranean diet components in local stores. This latter issue was specifically noted in studies from non-Mediterranean countries like the U.S., U.K., Australia, and Netherlands (Tsofliou et al., 2022). Additionally, the high cost of food like fresh fruits, vegetables, and fish was identified as a barrier (Tsofliou et al., 2022). Cost can be a significant barrier especially for people living in impoverished areas where there might already be limited food choices. A study assessing cost of Spanish graduate students to follow the Mediterranean diet or Western diet, a diet high in trans fats and refined sugars, found that the Mediterranean diet was more expensive (Lopez et al., 2009). Economic constraints can lead to consumption of less healthy diets. Similarly, barriers to the DASH diet include cost, poor cooking skills, and lack of support from household members (Tyson et al., 2023). In a study including low-income Latinos and African Americans with type 2 diabetes, participants reported lack of nutrition education was an impediment to make informed dietary choices and following a healthy diet (Bross et al., 2022). This indicates that people may not follow these diets not due to lack of will, but because they lack access, and the necessary tools and skills.

Furthermore, facilitators to the Mediterranean and DASH diets include increased awareness of the benefits of the diet, social support from family and friends, and integration of the diet items into traditional meals (Tsofliou et al., 2022). Family and friends can be crucial support as they can motivate people to continue to make better dietary choices (Ferranti et al., 2013; Laiou et al., 2020). Also, encouraging people to incorporate healthier items to their traditional meals might be easier than to encourage people to adopt a new dietary pattern (Tsofliou et al., 2022). Other facilitators to adopt healthy dietary patterns include having access to healthy foods, living alone or with supportive household member and having willpower and motivation for change (Tyson et al., 2023). Understanding individual and external factors that influence behaviors change is key when promoting the adoption of dietary patterns like the MIND diet.

The Capability, Opportunity, Motivation-Behavior (COM-B) model is at the core of an overarching framework called the Behavior Change Wheel, which is a three-stage approach to designing a behavior change intervention (Michie et al., 2011). The COM-B model states that behavior change involves modifying one or more of its components: capability, opportunity, and motivation (Michie et al., 2011). The model components are further divided which results in 6 total subcomponents: psychological capability, physical capability, physical opportunity, social opportunity, reflective motivation, and automatic motivation (Michie et al., 2011). These subcomponents were defined by Michie et al. (2011) as:

- *Capability* is a person's psychological and physical ability to engage in a specific behavior. Psychological ability relates to the thought process of engaging in a specific behavior for example knowledge and understanding. It can also be physical, for example, having the physical skills, strength, or stamina to perform the behavior.
- *Opportunity* reflects outside factors that make a behavior possible. Physical opportunity relates to environmental influences for a particular behavior and social opportunities relate to cultural factors influencing behavior.
- *Motivation* is a brain process that influences and directs behavior. Reflective motivation relates to evaluation, planning, and goals. Automatic motivation relates to emotions and impulses that arise from learning or innately.

The COM-B model has been used before to explore barriers and facilitators to physical activity in obese pregnant women (Bentley et al., 2019) and to explore barriers and facilitators to the adoption of the MIND diet in 40–50 year old men and women living in Northern Ireland and Italy (Timlin et al., 2021). There have not been studies assessing the relationship between the MIND diet and caregivers' health, but the MIND diet has the potential to improve caregivers' physical and psychological health. The aim of this study was to assess capability, opportunities, and motivation of informal female caregivers of people with ADRD to follow the MIND diet using the COM-B approach.

## Materials and methods

The iCARE study was a cross-sectional online survey study on informal female caregivers of people with ADRD.

Eligibility criteria to participate in iCARE included (1) identifying as women of at least 18 years of age; (2) providing at least 10 h of unpaid care per week to a family member or friend living in the community with ADRD.

Participants for the iCARE study were recruited using the Cloud Research Prime Panels online platform. Cloud research is an online platform where people across the U.S. register to participate in online survey research. Demographics of respondents in Cloud Research are diverse and comparable to U.S. adult population in terms of gender, age, race, ethnicity, and Census region (Chandler et al., 2019). Therefore, this type of online convenience sample can yield generalizable findings. Information about the present study was sent to potential participants living in the U.S through Cloud Research. Study information and survey was available in English and Spanish; however, all participants completed the survey in English. First, women were presented with the study title and identifying words for the study. After receiving the initial recruitment notification, interested individuals selected the study link which presented them with more information about the study and inclusion criteria. Those who decided to participate were then presented with the informed consent which they signed electronically before beginning the survey. This study was approved by the University of Illinois at Urbana-Champaign Institutional Review Board and informed consent was obtained from all participants.

The analytical sample of the present study included 299 caregivers (Table 1). Initially, 1,137 potential participants responded the inclusion criteria questions. Of those, 489 respondents did not meet the inclusion criteria. Respondents who met the inclusion criteria, 648, proceeded to read the study consent form and 459 participants provided electronic consent. Data from 78 participants was removed because the age of the care recipient was < 30. According to the National Institute on Aging, although rare, early-onset Alzheimer's can begin as early as 30 years of age. Data from other 82 participants was removed because they had a rate of completion of < 80% of the survey.

**Table 1:** Caregiver's characteristics.

	***n***	**% or *M* (SD)**
Age	297	37.7 (13.7)
BMI (Kg/m^2^)	277	28.3 (7.8)
Underweight (< 18.5)	16	5.8%
Normal weight (18.5–24.9)	99	35.7%
Overweight (25–29.9)	69	24.9%
With obesity (30)	93	33.6%
Education	299	
Less than high school	10	3.3%
High school diploma	107	35.8%
Some college or university	116	38.8%
Bachelor's degree	37	12.4%
Postgraduate	29	9.7%
Annual household income	299	
< $35,000	150	50.2%
$35,001–$75,000	99	33.1%
>$75,001	50	16.7%
Race	299	
White	215	71.9%
Black or African American	53	17.7%
American Indian or Alaskan Native	8	2.7%
Asian	10	3.3%
Native Hawaiian or Other Pacific Islander	1	0.3%
Other	12	4.0%
Ethnicity	281	
Non-Latino	241	85.8%
Latino	40	14.2%
Relationship to care recipient	298	
Daughter	92	30.9%
Granddaughter	45	15.1%
Wife	33	11.1%
Daughter in law	7	2.3%
Other relative	31	10.4%
Friend	90	30.2%

Information about caregivers' age, height, weight, education, income, race, and ethnicity was collected. Care recipient's demographic information was also collected including sex, age, and relationship to caregiver. Caregivers were asked to report on the care recipient's ADRD diagnosis by selecting from a list of possible diagnosis (Table 2).

**Table 2:** Care recipient's characteristics.

	***n***	**% or *M* (SD)**
Age	299	68.7 (15.0)
Sex	299	
Female	222	74.2%
Male	77	25.8%
Diagnosis	299	
Alzheimer's disease	126	42.4
Mixed dementia	62	20.9
Parkinson's disease	24	8.1
Dementia with Lewy bodies	15	5.1
Vascular dementia	14	4.7
Normal pressure hydrocephalus	8	2.7
Huntington's disease	7	2.4
Frontotemporal dementia	4	1.3
Creutzfeldt-Jakob disease	1	0.3
Wernicke-Korsakoff syndrome	1	0.3
No known diagnosis but experiencing memory difficulties and/or other concerning behavior(s) that may require care	35	11.8

Constructs of the COM-B model were assessed using a modified interview/focus group guide from Timlin et al. (2021) (see Supplementary material). Participants were asked the same questions as in the study of Timlin et al. (2021), but answers were presented in Likert scales and multiple choice. Participants received a short paragraph of information introducing them to the MIND diet and the MIND diet items before answering questions regarding the MIND diet. The questionnaire included at least one question for each of the 6 COM-B subcomponents: psychological capability, physical capability, social opportunity, physical opportunity, reflective motivation, and automatic motivation. Questions explored whether eating the MIND diet items was something participants were normally doing (e.g., 0 = never, 4 = always), confidence in cooking/eating the MIND diet (e.g., 0 = not confident at all, 4 = very confident), extent to which family would encourage/discourage the MIND diet (0 = not at all, 4 = very much), barriers, and facilitators to consume the MIND diet, and feelings about the MIND diet. When assessing barriers and facilitators participants were provided with a list and asked to select all the barriers and facilitators that applied to them. This list included: (1) access to food and stores, (2) transportation, (3) season, (4) budget or money, (5) time, (6) cooking skills, (7) culture, (8) family, (9) caregiving, and (10) friends. They were also given the option to add other barriers and facilitators. The list was created based on MIND diet barriers and facilitators reported by Timlin et al. Caregiving was added to the list as this relates specifically to the current sample.

### Analysis

Descriptive statistics were calculated for demographic data, including means and standard deviations for continuous variables and frequencies for categorical variables. Frequencies and percentages were computed to report survey questions results. All statistical analyses were performed with SPSS software, version 29.

Data from participants responding at the extremes as “Never” and “Always” to the questions, “To what extent is eating MIND diet, foods something you normally do? Do you eat MIND diet foods each day?” was assessed (*n* = 97) for differences between those who responded “rarely,” “sometimes,” and “often.” There were no statistically significant differences regarding age, education, and income for those who reported “never” and “always” and those responding “rarely,” “sometimes,” and “often” (*ps* > 0.05).

## Results

Caregivers' and care recipients' characteristics are detailed in Tables 1, 2. Briefly, participants were 299 female caregivers of people with ADRD who were on average 37.7 ± 13.7 years old (18–78 years old), White (72%), 58.5% with overweight or obesity, and had an annual household income < $35,000 dollars (50.2%). They provided care for a female (74.2%), on average 68.7 ± 15.0 years old (30–102 years old), and with Alzheimer's disease (42.6%).

### Capability and motivation

When asked about whether eating MIND diet foods is something they normally do, 8.4% of participants reported it was something they always do, 95% CI (0.05, 0.12) and 24.1% reported never eating MIND diet foods, 95% CI (0.19, 0.29). Only 11.4% of participants reported that friends and family will not at all support eating the MIND diet, 95% CI (0.08, 0.15). Whereas 67.1% of them reported that consuming the MIND diet would somewhat to very much be supported by friends and family, 95% CI (0.62, 0.72). Thirty-six percent of the participants also reported they were slightly confident or not at all confident to be able to eat or cook MIND diet items, 95% CI (0.31, 0.42). Fifty-seven percent of participants felt somewhat good or extremely good about eating the MIND diet, 95% CI (0.52, 0.63).

### Opportunity

**Figure 1:**
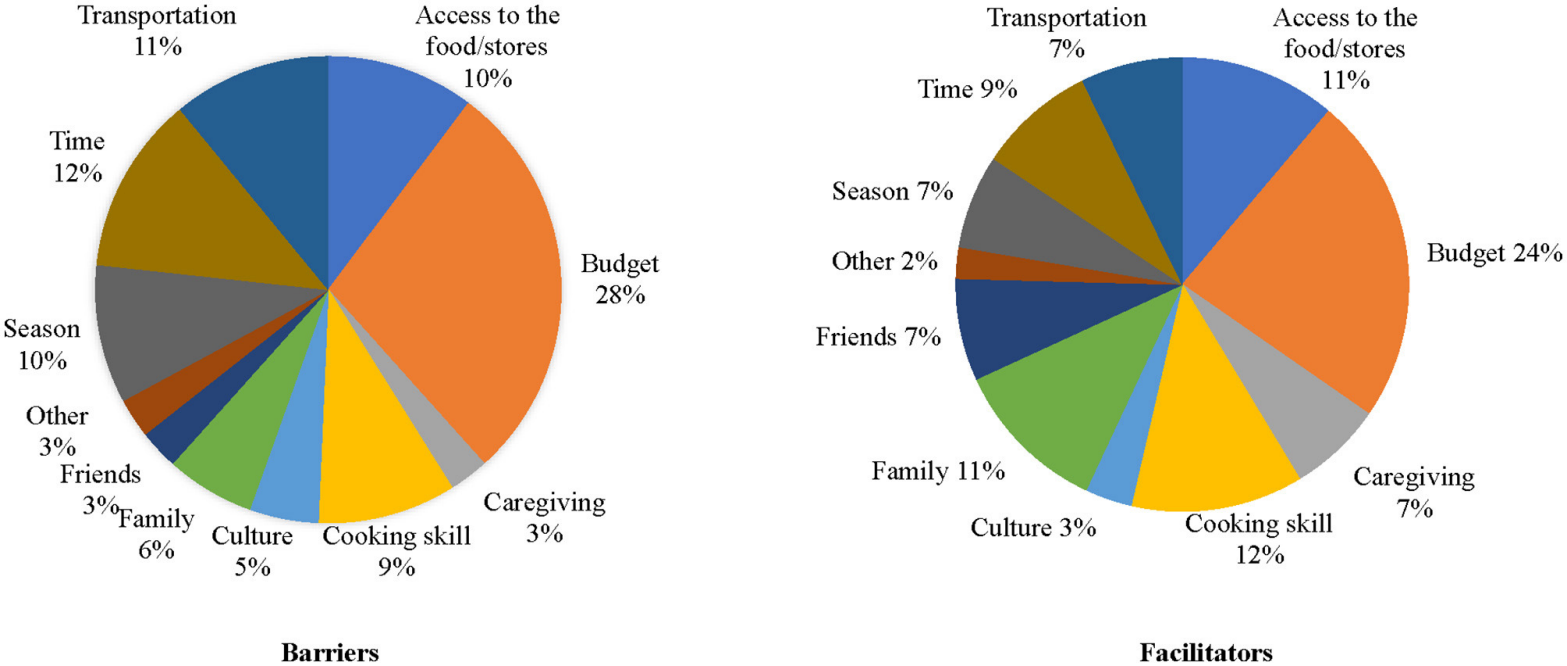
Barriers and facilitators to consuming the MIND diet.

In relation to friends and family, 56.9% of participants responded that their family or friends would somewhat to very much encourage them to eat MIND diet foods, 95% CI (0.52, 0.63) and 4.7% of participants responded that their family or friends would very much discourage them from eating the MIND diet, 95% CI (0.02, 0.07). The three most common barriers to the MIND diet were budget (28.0%), time (12.3%), and transportation (11.0%). The three most common facilitators to the MIND diet were budget (23.5%), cooking skills (12.3%) and access to food and stores (11.2%) or family support (11.2%; Figure 1).

## Discussion

The purpose of the present study was to assess capability, opportunities, and motivation of informal female caregivers of people with ADRD to follow the MIND diet using the COM-B approach. The study showed caregivers of people with ADRD have the motivation to consume the MIND diet as 57% of the sample felt somewhat good or extremely good about the MIND diet. Interventions that promote the MIND diet might be well received by this population. However, the results also showed that caregivers are not consuming the MIND diet. Less than 10 percent of the sample reported eating the MIND diet items regularly and about a quarter of the sample reported never eating the MIND diet items. These results are consistent with the typical American diet. According to the report for the 2020–2025 dietary guidelines for Americans, the typical American diet is too high in calories, contains added sugars saturated fats, and sodium (USDA and USDHHS, 2020). Americans also have low intake of vegetables, fruits, dairy, and whole grains, and the most consumed protein foods include beef, chicken, pork, processed meats, and eggs (USDA and USDHHS, 2020). Similar results have been reported in a study by Wang et al. (2019) where caregivers reported lack of healthy eating and constantly eating fast foods, sweets, and meat.

A third of the sample of caregivers in this study also reported low confidence or no confidence at all in eating or cooking the MIND diet items. Since eating some of the MIND diet items is not something they regularly do, caregivers might not feel confident in cooking these items. Health literacy and nutrition knowledge has the potential to influence nutrition patterns. The use of food labels has been positively associated with nutrition knowledge, vegetable consumption, and less sugar sweetened soda intake (Persoskie et al., 2017). In a community-based nutrition education program for adult men and women, there was a significant increase in fruit and vegetable consumption and decrease in fat intake after participating in a Full Plate Diet intervention (Downes et al., 2019). This intervention included a nutrition program to increase awareness of the consumption of nutrient-dense foods and high-fiber foods to increase satiety and reduce calorie intake for weight loss and thereby improve health. In another study, an intervention for caregivers to improve diet of people with dementia showed that increased nutritional knowledge for dementia significantly improved caregivers' healthy eating behavior (Hsiao et al., 2020).

The majority of the caregivers responded that their family and friends would encourage them to follow the MIND diet, and that they would not be discouraged by family or friends to consume the MIND diet items. Social support from family and friends to adopt new diets is important as eating can be a social activity and dietary practices can provide a sense of belonging to a certain group (Hendricks et al., 1988). In a previous study by Timlin et al. (2021) on barriers and facilitators to uptake the MIND diet, family support was reported as a barrier. In the present study family was cited as one of the top three facilitators to follow the MIND diet. Social networks have been positively associated with healthy eating and better diet quality in women (Mötteli et al., 2017; Shand et al., 2021) and for caregivers it can be a source of motivation.

Caregivers in this study reported budget as both a barrier and facilitator to the MIND diet. Several environmental factors influence nutrition including socio-economic status, access to healthful foods, and food security. According to a study by Jutkowitz et al. (2017), the total lifetime cost of care for a person with ADRD from diagnosis, at a mean age of 83, is $373,527 (2020 dollars). Families contribute about 70% of that amount in the form of unpaid care, medication, food, and other expenses (Jutkowitz et al., 2017). This has a great impact on the finances of caregivers as it is estimated that 41% of caregivers have a household income of $50,000 or less (Alzheimer's Association, 2023). In this study, half of the participants had an annual household income of < $35,000. Studies have shown that healthful foods cost more than less healthy options (Drewnowski, 2010; Wilde and Llobrera, 2009). Lower socio-economic status has also been associated with energy-dense, nutrient-poor diets, which contributes to poorer health (Darmon and Drewnowski, 2008). Food apartheid, referred to as the systemic and intentional inequities in food access and quality that disproportionately affect marginalized populations, contribute to food insecurity. In many minoritized neighborhoods across the U.S., there is a lack of grocery stores and finding healthy foods can be expensive and difficult (Grigsby-Toussaint et al., 2010). The combination of the economic cost of caregiving and low socio-economic status puts caregivers at higher risk of poor nutrition and health.

Caregivers also listed time as a major barrier to the MIND diet which is consistent with the barriers in the healthy eating literature (DiSantis et al., 2016; Welch et al., 2009; Yeh et al., 2008). In a study exploring fruit and vegetable consumption among a diverse multi-ethnic population in the U.S. perceived lack of time due to long working hours (Yeh et al., 2008). Furthermore, long time spent cooking has also been cited as a barrier to consuming a healthier diet (Yeh et al., 2008). Informal ADRD caregivers provided ~18 billion hours of unpaid care in 2022 (Alzheimer's Association, 2023). This is an average of 30 h per week based on average state minimum wage and average cost of a home health aide (Alzheimer's Association, 2023). Caregivers experiencing significant caregiving strain and those providing extensive care might have less time to prepare meals, leading them to choose unhealthy options like fast food (Wang et al., 2019). Based on the results from this study, caregivers have social opportunities to engage in the MIND diet as they report they would have support from family and friends to eat MIND diet items, but they lack physical opportunities including budget and time.

This study has some limitations. First, we did not collect information on dietary intake of caregivers. Therefore, we do not know whether caregivers were consuming the MIND diet items. Information from a food frequency questionnaire or a food diary is needed to understand which MIND diet items caregivers are already consuming, and which items need intervention. Second, the survey nature of the study did not allow for further elaboration on participant answers. For example, we were not able to assess if caregivers did not feel confident in eating and cooking the MIND diet items because of lack of access to the foods, their cooking skills, or other reasons. Future studies are needed to understand current MIND diet patterns of caregivers of people with ADRD. Another limitation is that our sample included only female caregivers. Data from the Alzheimer's Association shows one third of ADRD caregivers are men (Alzheimer's Association, 2023). Therefore, our findings may not be generalizable to male caregivers. Additionally, participants were recruited from an online platform only, therefore, this sample may not be representative of all caregivers. Future studies may consider including both male and female caregivers within online platforms and beyond to examine the MIND diet. Our study also had several strengths, including focusing on a population that is understudied. In nutrition-dementia research, the focus is often the person with ADRD, and few studies have included caregivers (Rabinowitz et al., 2007; Rullier et al., 2013, 2014; Torres et al., 2010; Wang et al., 2019). Nutrition and dietary patterns of the caregivers is an important issue as caregivers' nutrition can influence the health outcomes of the person with ADRD (Rullier et al., 2013, 2014). Another strength is that the study investigated a dietary pattern that has not been explored among caregivers. The MIND diet has the potential to benefit the physical and psychological health of caregivers.

MIND diet interventions for female caregivers of people with ADRD are needed. Components of social support should be incorporated in these interventions especially from family and friends (motivation) to aid in the adoption of new dietary patterns. There should also be a focus on budget planning and improving cooking skills (capability), as well as making MIND diet food items accessible (opportunity) to ADRD caregivers. Understanding how to increase capability, opportunities, and motivation for the MIND diet is needed to improve caregivers' health.

### Implications for public health

The findings in this study highlight significant public health implications. Caregivers face multiple challenges that negatively impact their health including poor dietary patterns. The MIND diet has the potential to improve caregiving-related health problems including depressive symptoms, stress related cognitive dysfunction, and poor sleep. Caregivers in this study reported they have the motivation to follow the MIND diet, however, they also report low consumption of MIND diet items. Public health initiatives should focus on increasing knowledge and skills related to healthy eating. Educational programs can provide strategies for making small changes and incorporating more MIND diet items into their diets as it has been shown that even adhering moderately to the MIND diet offers health benefits (Morris et al., 2015). Caregivers also reported budget and access to food/stores as a barrier to consuming the MIND diet. Public health policies must aim to increase accessibility to affordable, healthy food options, particularly for those in low-income and rural areas. Strategies can include providing subsidized access MIND diet foods, as well as partnerships with local businesses to ensure that items are available and affordable. Caring for a person with ADRD can have a great impact on the finances of caregivers. They contribute not only with unpaid care but also in other expenses like paying for housing, medication, and food. The combination of the economic cost of caregiving and low socio-economic status puts caregivers at higher risk of poor nutrition and health. Improving the health of caregivers through diet has the potential to improve the quality of life for both caregivers and the person they care for.

## Data Availability

The raw data supporting the conclusions of this article will be made available by the authors, without undue reservation.
