# Supplementary material for: The COM-B model: a cross-sectional survey assessing capability, opportunities, and motivation to follow the MIND diet among informal female caregivers of people with Alzheimer's disease and related dementias
Source: Front Dement. 2024 Oct 3;3:1451310. doi: 10.3389/frdem.2024.1451310 (PMC11484045; doi:10.3389/frdem.2024.1451310)
Supplement: Supplementary file 1 [file Data_Sheet_1.PDF]

MIND Diet Capability, Opportunities, and Motivation Questionnaire

Construct	Questions (modified from Timlin et al., 2021)
**Psychological Capability**	To what extent is eating MIND diet foods something you normally do?<br>(4) Always (3) Often (2) Sometimes (1) Rarely (0) Never
**Physical Capability**	To what extent are you confident in cooking/eating MIND diet foods?<br>(4) Very confident (3) Fairly confident (2) Somewhat confident (1) Slightly confident<br>(0) Not confident at all
**Social Opportunity**	To what extent would your family or friends encourage you to eat MIND diet foods?<br>(0) Not at all (1) A little bit (2) Somewhat (3) Quite a bit (4) Very much<br><br>To what extent do/would your family or friends discourage you from eating MIND diet foods?<br>(0) Not at all (1) A little bit (2) Somewhat (3) Quite a bit (4) Very much
**Physical Opportunity**	Is there anything in your work or/and home environment that might **help you** eat the MIND diet foods? What things will encourage you to consume the MIND diet? Select all that apply.<br>☐Access to the food/stores<br>☐Transportation<br>☐Season<br>☐Budget/Money<br>☐Time<br>☐Cooking skill<br>☐Culture<br>☐Family<br>☐Caregiving<br>☐Friends<br>☐Other<br>Is there anything in your work or/and home environment that might **discourage** you from eating the MIND diet foods? What are the challenges to consuming the MIND diet? Select all that apply.<br>☐Access to the food/stores<br>☐Transportation<br>☐Season<br>☐Budget/Money<br>☐Time<br>☐Cooking skill<br>☐Culture<br>☐Family<br>☐Caregiving<br>☐Friends<br>☐Other
**Reflective Motivation**	To what extent would eating the MIND diet be supported by your friends and family?<br>(0) Not at all (1) A little bit (2) Somewhat (3) Quite a bit (4) Very much
**Automatic Motivation**	How do you feel about eating the MIND diet?<br>(1) Extremely good (2) Somewhat good (3) Neither good nor bad (4) Somewhat bad<br>(5) Extremely bad
